# The end of Roe v. Wade: implications for Women’s mental health and care

**DOI:** 10.3389/fpsyt.2023.1087045

**Published:** 2023-05-05

**Authors:** Amalia Londoño Tobón, Eileen McNicholas, Camille A. Clare, Luu D. Ireland, Jennifer L. Payne, Tiffany A. Moore Simas, Rachel K. Scott, Madeleine Becker, Nancy Byatt

**Affiliations:** ^1^Department of Psychiatry, Georgetown University School of Medicine, Washington, DC, United States; ^2^UMass Chan Medical School, Worcester, MA, United States; ^3^Department of Obstetrics and Gynecology, Downstate Health Sciences University, Brooklyn, NY, United States; ^4^Department of Obstetrics and Gynecology, University of Massachusetts Chan Medical School, Worcester, MA, United States; ^5^Department of Psychiatry and Neurobehavioral Sciences, University of Virginia, Charlottesville, VA, United States; ^6^Department of Psychiatry, University of Massachusetts Chan Medical School, Worcester, MA, United States; ^7^Department of Pediatrics, University of Massachusetts Chan Medical School, Worcester, MA, United States; ^8^Department of Population & Quantitative Health Sciences, University of Massachusetts Chan Medical School, Worcester, MA, United States; ^9^MedStar Health Research Institute, Georgetown University School of Medicine, Washington, DC, United States; ^10^Departments of Psychiatry and Human Behavior, Sydney Kimmel Medical College, Integrative Medicine and Nutritional Sciences, Thomas Jefferson University, Philadelphia, PA, United States

**Keywords:** abortion, reproduction, Roe v Wade, Dobbs v Jackson, mental health, perinatal, psychiatry, obstetrics and gynecology

## Abstract

The Supreme Court decision in Dobbs v. Jackson in June 2022 reversed precedent which had previously protected abortion prior to fetal viability as a universal right within the United States. This decision almost immediately led to abortion restrictions across 25 states. The resulting lack of access to abortion care for millions of pregnant people will have profound physical and mental health consequences, the full effects of which will not be realized for years to come. Approximately 1 in 5 women access abortions in the U.S. each year. These women are diverse and represent all American groups. The Supreme court decision, however, will affect populations that have and continue to be marginalized the most. Forcing pregnant individuals to carry unwanted pregnancies worsens health outcomes and mortality risk for both the perinatal individual and the offspring. The US has one of the highest maternal mortality rates and this rate is projected to increase with abortion bans. Abortion policies also interfere with appropriate medical care of pregnant people leading to less safe pregnancies for all. Beyond the physical morbidity, the psychological sequelae of carrying a forced pregnancy to term will lead to an even greater burden of maternal mental illness, exacerbating the already existing maternal mental health crisis. This perspective piece reviews the current evidence of abortion denial on women’s mental health and care. Based on the current evidence, we discuss the clinical, educational, societal, research, and policy implications of the Dobbs v. Jackson Supreme Court decision.

## Introduction

Abortion is common—almost one in five pregnancies ended in abortion in 2014, and this number has been increasing (1, 2). In June 2022, in Dobbs v. Jackson Women’s Health Organization (“Dobbs decision”), the Supreme Court reversed the nationwide right to abortion care in the U.S. and returned decision-making power to state governments. Multiple medical societies, including the American Medical Association (AMA), the American Psychiatric Association (APA), and the American College of Obstetricians and Gynecologists (ACOG), denounced the ruling, as “a direct blow to bodily autonomy, reproductive health, patient safety, and health equity” (3–5). Given the ramifications of this decision, it is critical for healthcare professionals to understand (1) what is known about abortion in the U.S., (2) the impact of decreased access to abortion and denied abortion on women’s overall wellbeing and mental health, and (3) the implications for healthcare (see Table 1).

**Table 1:** Select organizations denouncing the Dobbs decision.

Professional organization	Statement denouncing Dobbs decision
American College of Obstetricians and Gynecologists	“Abortion is a safe, essential part of comprehensive health care, and just like any other safe and effective medical intervention, it must be available equitably to people, no matter their race, socioeconomic status, or where they reside” (5).
American Psychiatric Association	“By dismantling nearly 50 years of legal precedent, the Court has jeopardized the physical and mental health of millions of American women and undermined the privacy of the physician-patient relationship… today’s ruling will put many pregnant women and their families into life-threatening and/or traumatic situations” (4).
American Academy of Family Physicians	“…opposed any legislation or regulation that interferes in the confidential relationship between a patient and their physician and the provision of evidence-based patient care for any patient —and this decision will allow states to gravely interfere in that relationship…”*(from joint statement with AAP, ACOG, ACOP, and APA)* (6).
American Society for Reproductive Medicine	“Decisions about healthcare, particularly reproductive healthcare, should be made by patients and physicians, not by interest groups, religious organizations, politicians, pundits, or Supreme Court Justices” (7).
American Medical Association	“State restrictions that intrude on the practice of medicine and interfere with the patient-physician relationship leave millions with little or no access to reproductive health services while criminalizing medical care. Access to legal reproductive care will be limited to those with the sufficient resources, circumstances, and financial means to do so—exacerbating health inequities by placing the heaviest burden on patients from Black, Latinx, Indigenous, low-income, rural and other historically disadvantaged communities that already face numerous structural and systemic barriers to accessing health care” (3).
American Association of Pediatrics	“This decision carries grave consequences for our adolescent patients, who already face many more barriers than adults in accessing comprehensive reproductive healthcare services and abortion care” (8).

### History of abortion in the U.S.

In 1973, the U.S. Supreme Court codified the right to abortion under the constitutional protection of privacy in Roe v Wade (9). In response to an onslaught of state-level abortion restrictions, the court ruled in Planned Parenthood v. Casey that states could not enact legislation that would place an “undue burden” on the fundamental right to abortion. In June 2022, both decisions were overruled by the Supreme Court’s Dobbs decision. With this reversal, legislation of abortion returned to state governments. Within months, due to a combination of pre-Roe bans, “trigger laws” (abortion restrictions meant to be enacted with the overturning of Roe), and new legislation, half of U.S. states have severely restricted abortion access. At the present, 25 states have pre-viability abortion restrictions and, of those, 12 have enacted total bans (10). While some bans have been temporarily blocked by state courts, many remain in effect and prevent individuals from accessing abortion care.

### Abortion rates and statistics in the U.S.

In the U.S., approximately one-half of pregnancies are unplanned, and 18% of all pregnancies end in induced abortions (1, 11). Individuals of all reproductive ages and ethnic, racial and socioeconomic backgrounds access abortions. Most (60%) seeking abortion already have children and 58% have never previously had an abortion (2). Women who access abortion are more likely to be low-income, unmarried, and from a racially or ethnically minoritized populations (2, 12). Black women access abortions at three times the rate of white women (2). The most common reasons for seeking an abortion are 1) inability to financially support a child, 2) unpreparedness to have a/another child, and 3) an abusive or unsupportive partner (13). Three-quarters of women cite three or more reasons for seeking abortion care (14), underscoring the complexity of decision making and the desire to make the right decision for them and their families.

Prior to the Dobbs decision, nearly one in five women traveled 50 miles or more to access abortion care, and nearly one in ten women crossed state lines (15, 16). Since the passing of the Texas Senate Bill 8 (SB8), which bans abortion after the detection of embryonic cardiac activity, the number of pregnant individuals traveling out of Texas to obtain abortion care has increased ten-fold (17) (see Table 2).

**Table 2:** Abortion denial case examples.

AS	A.S. is a 30yo G4P3003 @ 13′3 with a pregnancy diagnosed with acrania at her routine ultrasound. The diagnosis is lethal. She resides in Missouri. Her physician recommends she travel to Illinois for an abortion. She works two minimum wage jobs and lives paycheck to paycheck as a single parent. She cannot afford the childcare, travel, or out-of-pocket cost to receive abortion care out of state.
LZ	L.Z. is a 44yo G3P1011 @ 7′6 weeks with an IUD in place. She has a history of peripartum cardiomyopathy with persistent left ventricular dysfunction. She is advised that continuing the pregnancy will put her at risk for heart failure or death. She asks for information about pregnancy termination, but this is not provided due to Texas restrictions on aiding or abetting abortion care as outlined in S.B. 8
EM	E.M. is a 22yo G1 @ 5′6 with an unintended pregnancy while taking oral contraceptive pills. She is a first-generation college student in Texas, hoping her pursuit of higher education will provide her family with newfound economic security. She is desperate to avoid the disruption of these plans. She is also in a relationship where she is experiencing abuse. She is willing to travel for an abortion but unsure of where to go or how to pay for care.

### Impact of denying abortions on perinatal morbidity and mortality

Pregnancy in and of itself is a physical state associated with increased morbidity and mortality, where continuing pregnancy is associated with a 14 times greater risk of death than abortion (18). In 2020, the U.S. maternal mortality rate reached 24 maternal deaths per 100,000 live births (2), making it the highest among industrialized nations (19, 20); these rates are substantially higher among marginalized communities (21). With a mortality rate of 0.4 deaths per 100,000 abortions, abortions conducted by trained specialists are far safer than pregnancy (19). Limiting access to abortion will increase the proportion of women continuing unwanted pregnancies and seeking “back alley” abortions by untrained individuals in suboptimal settings, (22) increasing both pregnancy and abortion related morbidity and mortality and exacerbating existing inequities in outcomes.

While the prevalence of self-managed medication abortion is growing, it has limitations. Despite numerous restrictions, the use at-home medication abortion allows many across the U.S. and the globe to continue to access abortion care (23). However, these medications are not 100% effective, safe for use in all situations, or effective later in pregnancy (24). About 2% of individuals undergoing medication abortion will require additional treatment (25) to safely complete the process. While self-managed medication abortions increase the availability of abortion care within U.S., this method is currently at risk of not being available to pregnant people, and is not a replacement for legal full-scope abortion care provided within the medical system. It is also important to note that in states in which legal abortion is unavailable, those who receive abortion care through these means and those that assist them may face criminal prosecution (26).

Abortion restrictions are associated with long-term morbidity. The most rigorous research evidence into the impact of abortion denial comes from the landmark Turnaway study, a prospective longitudinal study of those seeking abortion care, which examined the short and long-term outcomes of women who received abortion care versus those who sought but were denied abortion care. Nearly 1,000 women were recruited between 2008 and 2010 from 30 abortion facilities around the U.S. The study demonstrated that being denied an abortion and carrying an unwanted pregnancy is associated with higher risk of preeclampsia and postpartum hemorrhage, chronic pain, and poor overall health that persisted long after the pregnancy (27). Tragically, one woman in the study denied abortion later died from a postpartum infection (27). There were no cases of abortion-related deaths. Given the mortality and morbidity associated with unwanted pregnancies, these negative health impacts and maternal deaths are likely to rise as abortion restrictions increase.

### Impact of denying abortions on perinatal mental heath

The Dobbs ruling will also have repercussions on women’s mental health. Obstetric and mental health outcomes are inextricably linked. Unintended pregnancy is associated with perinatal depression and anxiety (28, 29). Conversely, the literature indicates that abortion does not lead to short-or long-term negative mental health outcomes, such as depression, anxiety, posttraumatic stress disorder, or suicide (30–35). Being denied abortion care is, however, associated with negative mental health outcomes. In the Turnaway Study, shortly after being denied an abortion, women reported heightened anxiety symptoms and lower self-esteem and life satisfaction compared to those who received an abortion. Additionally, women who have a prior history of a mental health condition or have experienced trauma are at greater risk of experiencing negative mental health outcomes when denied a wanted abortion (36, 37).

Abortion is also associated with significant social stigmatization in many communities across the U.S. In the Turnaway study, more than half of women seeking an abortion felt they would be looked down upon at least “a little bit” by people close to them if they knew they had sought an abortion. Higher perceived stigma about abortion is associated with higher odds of experiencing psychological distress over the 5-year follow up period (38). The anxiety that individuals experience after receiving abortion care appears to be more closely linked to social stigmatization than to personal regret. More than 95% of women felt that abortion was the correct decision for them and their families 5 years later (39). In this way the Dobbs decision may continue to worsen women’s mental health by furthering abortion stigma in some communities.

Intimate partner violence (IPV) is a leading reason for seeking abortion. As many as 22% of women seeking abortions have been exposed to recent violence from a partner, and those receiving abortions are able to extricate from abusive relationships more than those denied abortions (40). Continued exposure to IPV has short and long-term effects on the mental and physical health of those experiencing violence. IPV increases a woman’s risk for mental health conditions such as depression, anxiety and trauma related disorders (41, 42). IPV also has adverse effects on pregnancy outcomes and on the individuals and their children including, but not limited to maternal death and preterm birth (43–45). Currently, homicide is a leading cause of death of pregnant women in the U.S. (46). Forcing people to carry unwanted pregnancies conceived with abusive partners will prolong their exposure to IPV and endanger them and their families.

### Impact of denying abortions on child and family health

Apart from the direct effects on mental health, abortion denial impacts socioeconomic wellbeing and contributes to economic insecurity of pregnant persons, their children, and families. Pregnant people who are denied the ability to end a pregnancy suffer worse health, social and financial outcomes as do their families. Carrying a pregnancy and caring for a child comes with enormous time, emotional and financial investments that are unattainable for some. Financial distress, hardship, and limited financial resources are frequently cited reasons for seeking an abortion. In the Turnaway Study, 76% of those seeking abortion did not have enough money to cover living expenses, and 51% lived below the federal poverty level (47). Four years after being denied an abortion, women were three times more likely to be unemployed compared to those who received desired abortions. Overall, individuals have more debt, lower credit scores, and increased financial insecurity years after being denied an abortion compared to individuals who were able to obtain their desired abortion (47).

Being denied an abortion also negatively affects the development and wellbeing of a woman’s other children. In the Turnaway study, existing children of those denied an abortion had lower mean child development scores, particularly in expressive language and self-help, compared to children of those who received an abortion (48). Children born later to women who were able to receive their desired abortion had improved maternal bonding, and economic security as compared to children born to those who were denied an abortion.

Similar results have been found in studies outside of the U.S., including the Prague study, which followed the development and mental health of 220 children over 35 years whose mothers were denied an abortion compared to pair-matched children of those who did not seek an abortion. The study found significant differences in psychosocial development and mental health and wellbeing; children of those who were denied abortions had long-term negative psychosocial outcomes (49).

### Implications for obstetric care and training

Beyond the poor outcomes of unwanted pregnancies, the Dobbs decision has profoundly impacted obstetric providers’ medical decision making. Prior to Dobbs, in most states, pregnancy termination was a routine treatment option for a number of pregnancy complications. In the aftermath of the Dobbs decision, states have enabled civil and criminal penalties for abortion on both pregnant persons and their health care providers (26). Fear of prosecution has created medical “grey areas,” such as in cases of incomplete, septic, or threatened spontaneous abortions or ectopic pregnancies, in which indicated obstetric care is potentially delayed or withheld until there are clear signs of maternal health compromise. There is growing evidence of cases where women were subjected to clinical decompensation due to hemorrhage or infection to justify intervention and termination of pregnancy (50–52). ACOG has called attention to the anticipated rise in similar cases in their amicus briefs, citing grave concerns over the potential increase in morbidity and mortality (53). Restriction of abortion access has created a “chilling effect,” which renders obstetric providers hesitant to deliver lifesaving or health-preserving medical care (5). Recent data out of two Texas hospitals demonstrates an almost two-fold increase in pregnancy related morbidity among women with previable pregnancy complications as a result of SB8 (54).

The Dobbs decision and subsequent bans will also limit medical education and abortion training for medical students, residents, and fellows. The Accreditation Council for Graduate Medical Education (ACGME) requires access to abortion training for obstetrics and gynecology residency programs. Of the 286 accredited obstetrics and gynecology residency programs currently with residents, 128 (44.8%) are in states that have already banned or are likely to ban abortion after the overturning of Roe v Wade. In contrast, as recently as 2020, 92% of residents reported some access to this training (55). Of the 6,007 current obstetrics and gynecology residents, 2,638 (43.9%) are likely to lack or have severely reduced access to robust in-state abortion training post-Dobbs. Temporary solutions to address limitations in abortion training include travel rotations, remote learning, and simulation (55). While these training modalities will be crucial to continuing a broad scope of care for comprehensive family planning and abortion care across the U.S., they will not be equivalent to abortion training available prior to the Dobbs decision. This educational deficiency raises concerns about training the next generations of obstetricians and gynecologists, who may lack skills needed to provide lifesaving care for pregnant patients requiring emergent uterine evacuation for pregnancy complications, including miscarriages and hemorrhage, unrelated to abortion.

### Implications for psychiatric care and training

Given the effects of abortion denial on obstetric and mental health outcomes, the Dobbs decision will likely lead to increased demand for psychiatric care both during and after pregnancy. Unwanted pregnancy is a risk factor for antenatal depression and anxiety, as well as postpartum depression (56, 57). The mental health system in the U.S. is already in crisis, and unable to meet the current demand for psychiatric care. Prior to the Dobbs decision, psychiatrists and allied mental health professionals had and continue to have limited training in the management of mental health disorders during and after pregnancy (58). The number of Reproductive Psychiatry Fellowships is growing, but are still limited in the U.S., and have not yet been accredited by the ACGME (58). Given the mental health workforce shortage and potential increase in demand for mental health providers since Dobbs, there will be even less access to mental health care. Filling the maternal mental healthcare gap will increasingly fall on obstetrical, family medicine, and primary care providers.

Limited short-term options exist for addressing the mental health workforce issues. Perinatal Psychiatry Access Programs (“Access Programs”) are an evidence-based model that increase the capacity of providers serving perinatal individuals to address their mental health needs (59). Investments in adaptations to Access Programs and other models of care will be needed to address the needs of women and children with psychiatric illness due to abortion restrictions. Additionally, psychiatric and other mental health professional organizations will need to increase (1) resources to support the mental health needs of women denied abortions, and (2) training for psychiatrists in reproductive and perinatal mental health. Recognizing Reproductive Psychiatry as an essential component in all psychiatric training, and as an ACGME recognized subspecialty, it will be crucial to building a workforce that is knowledgeable in addressing the needs of women being denied abortions.

### The Dobbs decision as a political determinant of health that will exacerbate health inequities

Political determinants of health (PDOH) are the ways in which policies and politics influence the social conditions that impact health outcomes. PDOH systematically structure relationships, distribute resources, and administer power to mutually reinforce or influence opportunities to advance health equity or exacerbate health inequities (60). Often the connection between social determinants of health (SDOH) and their political roots are lost, but the overturning of Roe v. Wade will be an unwelcome flagship example of a PDOH driving health inequities in the U.S.

Criminalization of abortion for pregnancy-capable individuals and their children will have other far-reaching implications with a greater impact for populations that have been marginalized in the U.S. Prior to the Dobbs decision, Black women and those with lower incomes accessed abortions at a higher rate than other communities. Following the Dobbs decision, marginalized communities will have less access to abortion care and will face greater challenges in accessing care out of state than their privileged counterparts. Those without resources will be forced to either (1) carry an unwanted pregnancy and have a forced birth while also bearing the burden of the social, obstetric, and mental health sequelae previously outlined, (2) undergo self-managed abortions, placing them at risk for arrest and prosecution when presenting for subsequent emergency care, and (3) in some cases, access unsafe abortions with increased risk of morbidity and mortality. Restrictions stand to worsen the health inequities, in addition to inequity within the U.S. criminal justice system, for Black and Brown individuals (61).

Given the disproportionate burden of stress associated with the unequal distribution of resources, marginalized individuals including but not limited to Black, Indigenous, Latinx, immigrants, refugees, sexual, gender and religious minoritized groups, will be more affected by the Dobbs decision. (62). The maternal mortality and mental health crises are already disproportionally negatively affecting minoritized individuals who have less access to comprehensive reproductive and overall health care (63). Additionally, these groups have historically been more impacted by abortion restriction-related clinic closures including having limited resources to travel long distances to receive care (64, 65). The Dobbs decision will add to the existing historical trauma that these populations have experienced and continue to experience due to structural racism and oppression.

## Conclusion

The Dobbs decision, which overturned Roe v. Wade, has serious implications for all women and childbearing individuals, as well as their children, families and generations to come. It affects obstetric care providers, mental health professionals, and the patients they serve in an already strained health system. It will increase demand for services where there are already limited providers and health care deserts. It will force providers to be reactive rather than proactive in caring for women and families. As a political determinant of health, this decision will disproportionally affect populations who have been historically, and continue to be, under-served and marginalized.

System-wide interventions to increase the capacity of obstetric and mental health professionals to respond to the mental and physical health and other effects that will arise from denied abortion will be critical to mitigation. Ongoing studies that continue to document the impact of this decision will be important to inform policy decisions. Advocacy is imperative to increase awareness of evidence-based information about the harmful effects of abortion denial and inform policy makers. The data is clear; women and families do better when they retain the ability to access safe abortion care. To realize a vision of healthy and resilient families in the U.S., we must ensure pregnant individuals have access to evidence-based reproductive health and abortion health care.

### Statement on language

We have used the term women in this piece to refer to pregnancy capable individuals and not to refer to gender identity. We recognize that not all pregnancy capable and childbearing people identify as women, and that *all* individuals, and especially those with the potential for childbearing, are impacted by the restriction of abortion in the U.S.

## Data availability statement

The original contributions presented in the study are included in the article/supplementary material, further inquiries can be directed to the corresponding author.

## Disclosures

The contents and views in this manuscript are those of the authors only. JP holds two patents: “Epigenetic Biomarkers of Postpartum Depression” and “Epigenetic Biomarkers of Premenstrual Dysphoric Disorder and SSRI Response.” She has received consulting fees from SAGE Therapeutics, Biogen, Merck, Brii Biologics, Pure Tech, Flo Health and Merck. She receives research funding from Janssen Pharmaceuticals. She has received honoraria from Global Learning Collaborative, CMEToGO, UPtoDate, and Karuna Therapetics. She owns Founder’s Stock options from Dionysus Digital Health. TMS is a consultant to Massachusetts Child Psychiatry Access Program for Moms (MCPAP for Moms) which is funded through the Massachusetts Department of Mental Health and administrated through Beacon Health Options. She is Medical Director of Lifeline for Moms at UMass Chan Medical School. RKS receives research funding through investigator sponsored research awards from Gilead Science and ViiV healthcare to study HIV and HIV prevention (awards managed by MedStar Health Research Institute). NB has received salary and/or funding support from Massachusetts Department of Mental Health via the Massachusetts Child Psychiatry Access Program for Moms (MCPAP for Moms). She is also the statewide Medical Director of MCPAP for Moms and the Executive Director of the Lifeline for Families Center at UMass Chan Medical School. She has served on the Medscape Steering Committee on Clinical Advances in Postpartum Depression. She received honoraria from Global Learning Collaborative, Medscape, and Mathematica. She has also served as a consultant for The Kinetix Group and JBS International.

## Author contributions

AT and EM completed initial draft of the manuscript. All authors critically reviewed and edited drafted for important intellectual content and provided approval for publication and agree to be accountable for all aspects of the work. All authors provided substantial contributions to the conception or design of the manuscript.

## Conflict of interest

The authors declare that the research was conducted in the absence of any commercial or financial relationships that could be construed as a potential conflict of interest.

## Publisher’s note

All claims expressed in this article are solely those of the authors and do not necessarily represent those of their affiliated organizations, or those of the publisher, the editors and the reviewers. Any product that may be evaluated in this article, or claim that may be made by its manufacturer, is not guaranteed or endorsed by the publisher.

## References

[1] Jones RK Witwer E Jerman J. AbortiPon incidence and service availability in the United States. New York: Guttmacher Institute (2019).

[2] Walensky RP Bunnell R Layden J Kent CK Gottardy AJ Leahy MA. Morbidity and mortality weekly report Centers for Disease Control and Prevention MMWR editorial and production staff (serials) MMWR editorial board. (2019).

[3] Resneck J. Dobbs rPuling is an assault on reproductive health, safe medical practice. (2022) available at: https://www.ama-assn.org/about/leadership/dobbs-ruling-assault-reproductive-health-safe-medical-practice. (Accessed December 10, 2022).

[4] American Psychiatric Association. American Psychiatric Association statement on Dobbs v. Jackson Women’s health Organization (2022) available at: https://www.psychiatry.org/News-room/News-Releases/APA-Statement-on-Dobbs-v-Jackson. (Accessed December 10, 2022).

[5] Hoskins IA. ACOG statement on the decision in Dobbs V. Jackson (2022) available at: https://www.acog.org/news/news-releases/2022/06/acog-statement-on-the-decision-in-dobbs-v-jackson. (Accessed December 10, 2022).

[6] American Academy of Family Physicians. Physicians: SCOTUS decision jeopardizes patient-physician relationship. Washington, D.C.: Penalizes Evidence-Based Car (2022).

[7] Cedars M. The Dobbs decision: a Statement from ASRM. (2022). available at: https://www.asrm.org/news-and-publications/news-and-research/announcements/the-dobbs-decision-a-statement-from-asrm/. (Accessed November 1, 2022).

[8] Szilagyi M. AAP statement on supreme court decision in Dobbs v. Jackson Women’s health Organization (2022). Available at: https://www.aap.org/en/news-room/news-releases/aap/2022/aap-statement-on-supreme-court-decision-in-dobbs-v.-jackson-womens-health-organization/. (Accessed November 1, 2022).

[9] Roe V. Wade, 410 U.S. 113 (1973). JUSTIA Available at: https://supreme.justia.com/cases/federal/us/410/113/. (Accessed November 1, 2022).

[10] Guttmacher Institute. State Bans on Abortion Throughout Pregnancy. Guttmacher Institute (2022) Available at: https://www.guttmacher.org/state-policy/explore/state-policies-later-abortions. (Accessed October 23, 2022).

[11] Guttmacher Institute. Fact sheet. Unintended Pregnancy in the United States. New York: Guttmacher Institute (2016) Available at: https://www.guttmacher.org/sites/default/files/factsheet/fb-unintended-pregnancy-us_0_4.pdf.

[12] Jerman J Jones RK Onda T. Characteristics of U.S. Abortion patients in 2014 and changes Since 2008. New York: Guttmacher Institute (2016).

[13] Biggs MA Gould H Foster DG. Understanding why women seek abortions in the US. BMC Womens Health. (2013) 13, 1–13. doi: 10.1186/1472-6874-13-29, PMID: 23829590 PMC3729671

[14] Finer LB Frohwirth LF Dauphinee LA Singh S Moore AM Reasons U.S. Women Have abortions: quantitative and qualitative perspectives. Perspect Sex Reprod Health. (2005) 37:110–8. doi: 10.1363/3711005, PMID: 16150658

[15] Fuentes L Jerman J. Distance traveled to obtain clinical abortion Care in the United States and Reasons for clinic choice. J Women's Health. (2019) 28:1623–31. doi: 10.1089/jwh.2018.7496 PMC6919239 31282804

[16] Maddow-Zimet I Kost K. Even Before Roe was Overturned, nearly one in 10 people obtaining an abortion traveled across state lines for care. New York: Guttmacher Institute (2022).

[17] White K Dane’el A Vizcarra E Dixon L Lerma K Beasley A.. Out-of-state travel for abortion following implementation out-of-state travel for abortion following implementation of Texas Senate Bill 8 of Texas Senate Bill 8. Texas Policy Evaluation Project. Available at: https://sites.utexas.edu/txpep/files/2022/03/TxPEP-out-of-state-SB8.pdf.

[18] Raymond EG Grimes DA. The comparative safety of legal induced abortion and childbirth in the United States. Obstet Gynecol. (2012) 119:215–9. doi: 10.1097/AOG.0b013e31823fe923, PMID: 22270271

[19] Hoyert LD. Maternal mortality rates in the United States, 2019. Atlanta, Georgia: National Center for Health Statistics (2021).

[20] Douthard RA Martin IK Chapple-McGruder T Langer A Chang S. Maternal mortality within a global context: historical trends, current state, and future directions. J Women's Health. (2021) 30:168–7. doi: 10.1089/jwh.2020.8863, PMID: 33211590 PMC8020556

[21] MacDorman MF Declercq E Thoma ME. Trends in maternal mortality by sociodemographic characteristics and cause of death in 27 states and the District of Columbia. Obstet Gynecol. (2017) 129:811–8. doi: 10.1097/AOG.0000000000001968, PMID: 28383383 PMC5400697

[22] Cohen SA. Facts and consequences: Legality, incidence and safety of abortion worldwide. (2009). Available at: https://www.guttmacher.org/sites/default/files/article_files/gpr120402.pdf. (Accessed October 24, 2022).

[23] Jones RK Donovan MK. Self-managed abortion may be on the rise, but probably not a significant driver of the overall decline in abortion. New York: Guttmacher institute (2019) Available at: https://www.guttmacher.org/article/2019/11/self-managed-abortion-may-be-rise-probably-not-significant-driver-overall-decline.

[24] American College of Obstetricians and Gynecologists. Medication Abortion Up to 70 Days Gestation. ACOG practice bulletin no. 225. Obstetr Gynecol. (2020) 136:e31–47. 10.1097/AOG.0000000000004082 32804884

[25] Upadhyay UD Desai S Zlidar V Weitz TA Grossman D Anderson P. Incidence of emergency department visits and complications after abortion. Obstet Gynecol. (2015) 125:175–3. doi: 10.1097/AOG.0000000000000603 25560122

[26] Gerdts C Dobkin L Foster DG Schwarz EB. Side effects, physical health consequences, and mortality associated with abortion and birth after an unwanted pregnancy. Womens Health Issues. (2016) 26:55–9. doi: 10.1016/j.whi.2015.10.001 26576470

[27] Liu CH Tronick E. Rates and predictors of postpartum depression by race and ethnicity: results from the 2004 to 2007 New York city PRAMS survey (pregnancy risk assessment monitoring system). Matern Child Health J. (2013) 17:1599–10. doi: 10.1007/s10995-012-1171-z, PMID: 23095945

[28] Ghaedrahmati M Kazemi A Kheirabadi G Ebrahimi A Bahrami M. Postpartum depression risk factors: a narrative review. J Educ Health Promot. (2017) 6:60. doi: 10.4103/jehp.jehp_9_16, PMID: 28852652 PMC5561681

[29] Robinson GE Stotland NL Nadelson CC. Abortion and mental health: guidelines for proper scientific conduct ignored. Br J Psychiatry. (2011) 200:78. doi: 10.1192/bjp.200.1.78, PMID: 22215872

[30] Stotland NL. Induced abortion and adolescent mental health. Curr Opin Obstet Gynecol. (2011) 23:340–3. doi: 10.1097/GCO.0b013e32834a93ac 21836505

[31] Steinberg JR Finer LB. Coleman, Coyle, Shuping, and rue make false statements and draw erroneous conclusions in analyses of abortion and mental health using the national comorbidity survey. J Psychiatr Res. (2012) 46:407–8. doi: 10.1016/j.jpsychires.2012.01.019, PMID: 22348853 PMC3699180

[32] Biggs MA Neuhaus JM Foster DG. Mental health diagnoses 3 years after receiving or being denied an abortion in the United States. Am J Public Health. (2015) 105:2557–63. doi: 10.2105/AJPH.2015.302803 26469674 PMC4638270

[33] Biggs A Rowland B Mcculloch CE Foster DG. Does abortion increase women’s risk for post-traumatic stress? Findings from a prospective longitudinal cohort study. Reprod Health. (2016) 6:9698. doi: 10.1136/bmjopen-2015 PMC4746441 26832431

[34] Biggs MA Gould H Barar RE Foster DG. Five-year suicidal ideation trajectories among women receiving or being denied an abortion. Am J Psychiatr. (2018) 175:845–2. doi: 10.1176/appi.ajp.2018.18010091 29792049

[35] Major B Appelbaum M Beckman L Dutton MA Russo NF West C. Abortion and mental health: evaluating the evidence. Am Psychol. (2009) 64:863–07. doi: 10.1037/a0017497, PMID: 19968372

[36] Wisner KL Appelbaum PS. Abortion restriction and mental health. JAMA Psychiat. (2023). Advance online publication. doi: 10.1001/jamapsychiatry.2022.4962, PMID: 36753289

[37] Biggs MA Brown K Foster DG. Perceived abortion stigma and psychological well-being over five years after receiving or being denied an abortion. PLoS One. (2020) 15:e0226417. doi: 10.1371/journal.pone.0226417, PMID: 31995559 PMC6988908

[38] Rocca CH Samari G Foster DG Gould H Kimport K. Emotions and decision rightness over five years following an abortion: an examination of decision difficulty and abortion stigma. Soc Sci Med. (2020) 248:112704. doi: 10.1016/j.socscimed.2019.112704, PMID: 31941577

[39] Roberts SCM Biggs MA Chibber KS Gould H Rocca CH Foster DG. Risk of violence from the man involved in the pregnancy after receiving or being denied an abortion. BMC Med. (2014) 12:144. doi: 10.1186/s12916-014-0144-z, PMID: 25262880 PMC4182793

[40] Devries KM Mak JY Bacchus LJ Child JC Falder G Petzold M. Intimate partner violence and incident depressive symptoms and suicide attempts: a systematic review of longitudinal studies. PLoS Med. (2013) 10:1–11. doi: 10.1371/journal.pmed.1001439 PMC3646718 23671407

[41] Bacchus LJ Ranganathan M Watts C Devries K. Recent intimate partner violence against women and health: a systematic review and meta-analysis of cohort studies. BMJ Open. (2018) 8:e019995. doi: 10.1136/bmjopen-2017-019995 PMC6067339 30056376

[42] Pastor-Moreno G Ruiz-Pérez I Henares-Montiel J Petrova D. Intimate partner violence during pregnancy and risk of fetal and neonatal death: a meta-analysis with socioeconomic context indicators. Am J Obstet Gynecol. (2020) 222:123–133.e5. doi: 10.1016/j.ajog.2019.07.045, PMID: 31394067

[43] Stover CS Tobon AL McFaul C Gorio MCF. A conceptual understanding of intimate partner violence behaviors in men: implications for research and intervention. Aggress Violent Behav. (2022) 65:101763. doi: 10.1016/j.avb.2022.101763

[44] Hill A Pallitto C McCleary-Sills J Garcia-Moreno C. A systematic review and meta-analysis of intimate partner violence during pregnancy and selected birth outcomes. Int J Gynecol Obstet. (2016) 133:269–6. doi: 10.1016/j.ijgo.2015.10.023, PMID: 27039053

[45] Lawn RB Koenen KC. Homicide is a leading cause of death for pregnant women in US. BMJ. (2022) 379:2499. doi: 10.1136/bmj.o2499, PMID: 36261146

[46] Foster DG Ralph LJ Biggs MA Gerdts C Roberts SCM Glymour MA. Socioeconomic outcomes of women who receive and women who are denied wanted abortions in the United States. Am J Public Health. (2018) 108:407–3. doi: 10.2105/AJPH.2017, PMID: 29345993 PMC5803812

[47] Foster DG Raifman SE Gipson JD Rocca CH Biggs MA. Effects of carrying an unwanted pregnancy to term on Women’s existing children. J Pediatr. (2018) 205:183–189.e1. doi: 10.1016/j.jpeds 30389101

[48] David HP. Born unwanted: mental health costs and consequences. Am J Orthopsychiatry. (2011) 81:184–2. doi: 10.1111/j.1939-0025.2011.01087.x 21486260

[49] Gonzalez O. How states enforce anti-abortion laws. Axios (2022) Available at: https://www.axios.com/2022/06/08/abortion-bans-penalty-fines-prison-us-states. (Accessed October 25, 2022).

[50] Feibel C. Because of Texas abortion law, her wanted pregnancy became a medical nightmare. National Public Radio (2022) Available at: https://www.npr.org/sections/health-shots/2022/07/26/1111280165/because-of-texas-abortion-law-her-wanted-pregnancy-became-a-medical-nightmare. (Accessed October 24, 2022).

[51] Belluck P. They had miscarriages, and new abortion Laws obstructed treatment. (2022). Available at: https://www.nytimes.com/2022/07/17/health/abortion-miscarriage-treatment.html. (Accessed October 24, 2022).

[52] Zurawski A. My pregnancy vs. the State of Texas. The Meteor (2022) Available at: https://wearethemeteor.com/texas-abortion-ban-stopped-doctors-helping-woman-miscarrying/.

[53] Dobbs v. J. Brief amicus curiae of American College of Obstetricians and Gynecologists American Medical Association American Academy of Family Physicians American Academy of Nursing American Academy of Pediatrics American Academy of Public Health Physicians. ACOG. (2021) Available at: https://www.acog.org/-/media/project/acog/acogorg/files/advocacy/amicus-briefs/2021/20210920-dobbs-v-jwho-amicus-brief.pdf?la=en&hash=717DFDD07A03B93A04490E66835BB8C5

[54] Nambiar A Patel S Santiago-Munoz P Spong CY Nelson DB. Maternal morbidity and fetal outcomes among pregnant women at 22 weeks’ gestation or less with complications in 2 Texas hospitals after legislation on abortion. Am J Obstetr Gynecol. (2022) 227:648–650.e1. 10.1016/j.ajog.2022.06.060 35803323

[55] Vinekar K Karlapudi A Nathan L Turk JK Rible R Steinauer J. Projected implications of overturning roe v Wade on abortion training in U.S. obstetrics and gynecology residency programs. Obstet Gynecol. (2022) 140:146–9. doi: 10.1097/AOG.0000000000004832, PMID: 35852261

[56] Biaggi A Conroy S Pawlby S Pariante CM. Identifying the women at risk of antenatal anxiety and depression: a systematic review. J Affect Disord. (2016) 191:62–77. doi: 10.1016/j.jad.2015.11.014 26650969 PMC4879174

[57] Mercier RJ Garrett J Thorp J Siega-Riz AM. Pregnancy intention and postpartum depression: secondary data analysis from a prospective cohort. BJOG. (2013) 120:1116–22. doi: 10.1111/1471-0528.12255 23651010 PMC3708972

[58] Osborne LM MacLean JV Barzilay EM Meltzer-Brody S Miller L Yang SN. Reproductive psychiatry residency training: a survey of psychiatric residency program directors. Acad Psychiatry. (2018) 42:197–1. doi: 10.1007/s40596-017-0672-x, PMID: 28194683

[59] Byatt N Simas TAM Lundquist RS Johnson J Ziedonis DM. Strategies for improving perinatal depression treatment in north American outpatient obstetric settings. J Psychosom Obstet Gynecol. (2012) 33:143–1. doi: 10.3109/0167482X.2012.728649, PMID: 23194018

[60] Dawes DE. Managing America’s crises means addressing the political determinants of health. Grantmakers in Health (2020) Available at: https://www.gih.org/views-from-the-field/managing-americas-crises-means-addressing-the-political-determinants-of-health/. (Accessed December 10, 2022).

[61] Bailey ZD Krieger N Agénor M Graves J Linos N Bassett MT. America: Equity and equality in health 3 structural racism and health inequities in the USA: Evidence and interventions. (2017). Available at: www.thelancet.com/. (Accessed November 1, 2022). 10.1016/S0140-6736(17)30569-X 28402827

[62] Lim D. (2020) I’m embracing the term ‘people of the global majority’ | by Daniel Lim | medium. Medium available at: https://regenerative.medium.com/im-embracing-the-term-people-of-the-global-majority-abd1c1251241 (Accessed November 1, 2022).

[63] Bryant AS Worjoloh A Caughey AB Washington AE. Racial/ethnic disparities in obstetric outcomes and care: prevalence and determinants. Am J Obstet Gynecol. (2010) 202:335–3. doi: 10.1016/j.ajog.2009.10.864, PMID: 20060513 PMC2847630

[64] Goyal V Brooks IHML Powers DA. Differences in abortion rates by race–ethnicity after implementation of a restrictive Texas law. Contraception. (2020) 102:109–4. doi: 10.1016/j.contraception.2020.04.008, PMID: 32304767 PMC7473327

[65] Artiga S Hill L Ranji U Gomez I. What are the implications of the overturning of roe v. Wade for racial disparities? Kaiser Family Foundation (2022) available at: https://www.kff.org/racial-equity-and-health-policy/issue-brief/what-are-the-implications-of-the-overturning-of-roe-v-wade-for-racial-disparities/# (Accessed January 25, 2022).

